# Model development to improve primary care services using an innovative network of homecare providers (WinCare) to promote blood pressure control among elderly patients with noncommunicable diseases in Thailand: a prospective cohort study

**DOI:** 10.1186/s12875-022-01648-4

**Published:** 2022-03-06

**Authors:** Boonsub Sakboonyarat, Mathirut Mungthin, Panadda Hatthachote, Yupaporn Srichan, Ram Rangsin

**Affiliations:** 1 Department of Military and Community Medicine, Phramongkutklao College of Medicine, Bangkok, Thailand; 2 Department of Pharmacology, Phramongkutklao College of Medicine, Bangkok, Thailand; 3 Department of Physiology, Phramongkutklao College of Medicine, Bangkok, Thailand; 4 Office of Disease Prevention and Control Region 1, Chiang Mai, Thailand

**Keywords:** WinCare, Homecare providers, Controlled blood pressure, Hypertension, Elderly patients, HRQoL, Thailand

## Abstract

**Background:**

Thailand has been rapidly approaching an aging society in conjunction with an increase in noncommunicable diseases (NCDs) especially hypertension and type 2 diabetes. Demographics and epidemiologic transitions create several challenges to the health system in Thailand in the case of long term care policies, in particular, modality to support home care. Therefore, the model development to facilitate primary care home services for elderly patients with NCDs using an innovative network of homecare providers (WinCare) was established. The study aimed to evaluate the effectiveness of WinCare to improve blood pressure (BP) control as well as health-related quality of life (HRQoL) among elderly patients with NCDs.

**Methods:**

A prospective cohort study was conducted between July 2019 and January 2020 in a suburban area, Chiang Mai Province, Thailand. The intervention included WinCare providers and WinCare application. WinCare provided check-in visits to measure subjects blood pressure and body weight (once weekly), played the roles of other home caregivers for the patients and recorded measurements and activities in the WinCare app for 6 months. The primary outcomes of the study were differences in systolic BP, diastolic BP, and controlled BP (systolic BP < 140 mmHg and diastolic BP < 90 mmHg) at 6-month follow-up between the intervention and control groups, adjusting for age, sex, marital status, comorbidities, alcohol consumption and smoking status.

**Results:**

A total of 104 subjects were initially recruited. Of the remaining 98 individuals, 52 were allocated to the intervention group and 46 to the control group. After adjusting baseline characteristics, no association existed between decreasing average systolic BP and intervention groups. However, diastolic BP of patients in the intervention group was on average 5.19 mmHg (95% CI -8.22, − 2.17) lower compared than that of the control group at 6-month follow-up. Furthermore, compared with patients in the control group, those in the intervention group were more likely to control BP, (AOR 3.03; 95% CI 1.02–9.01) at 6-month follow-up.

**Conclusion:**

Establishing a network of homecare providers (WinCare) was feasible in a community setting. This innovative network was able to facilitate elderly patients with NCDs residing in a suburban community to improve BP control at least at 6-month follow-up.

**Trial registration:**

Trial identification number was TCTR20200312007, First submitted date:12/03/2020.

## Background

At present, the contextual shifts including demographic and epidemiologic transitions create several challenges to health care systems in Thailand. From 2017 to 2100, Thailand was forecasted to have population declines greater than one half [1]. Therefore, Thailand is considered one of the world’s promptly aged societies [2]. The United Nations Population Fund Thailand reported that an increasing trend of individuals living alone has occurred in approximately 6% of total households in 1987 to 14% in 2013. In the next two decades, it has been predicted that one in five households will constitute one-person households [3, 4]. In addition, the prevalence of elderly patients with noncommunicable diseases (NCDs) in Thailand are more likely to increase [5, 6]. Hypertension (HT) is the most common NCDs in Thailand. The Thai National Health Examination Survey in 2014 demonstrated that one of four Thais had HT [7]; additionally, one fourth of Thai patients with HT could not control blood pressure (BP) [8]. Uncontrolled BP leads to serious complications including ischemic heart disease, stroke and renal insufficiency [8–11].

In Thailand, primary care in the community involves health care services under the health promoting hospital located in that area and provided by village health volunteers; however, human resources are limited compared with the responsibility of care for a large number of patients in the community. Furthermore, recent evidence in 2019 confirmed that nurses were likely to be in critical shortage by 2026 [12, 13]. The contextual changes contributing the challenges in the Thai health care system in the case of long term care policies, in particular modality of care, include training and support to home care. The model development of primary care services for elderly patients using an innovative network of homecare providers (WinCare) was established to provide additional human resources to deliver nonmedical care which filled the gaps among health care providers, primary care units and patients residing in a community. Moreover, this innovation model will contribute rewarding jobs with additional remuneration for human resources in communities. The study aimed to evaluate the effectiveness of model development of primary care services using an innovative network of homecare providers (WinCare) to improve BP control as well as health-related quality of life (HRQoL) among elderly patients.

## Methods

### Study design and participants

A prospective cohort study was conducted between July 2019 and January 2020 in Nong-Hoi Community, Mueng Chiang Mai District, Chiang Mai Province (northern Thailand, a suburban area 680 km from Bangkok). The eligibility criteria for participants included (i) patients with type 2 diabetes (T2D) and/or HT aged at least 60 years, (ii) residing in the authorized area of Nong-Hoi Health Promoting Hospital, Mueng Chiang Mai District, Chiang Mai Province (iii) willing to participate in the study and providing written informed consent and (iv) required nonmedical home care providers. The exclusion criteria are described below.

(i)  bedridden patients
(ii) end stage renal disease with renal replacement therapy or eGFR < 30 ml/min/1.73m^2^
(iii) a history of myocardial infraction 6 months previously
(iv) a history of heart failure; New York Heart Association (NYHA) ≥ class III
(v) participating in other clinical controlled trials

These criteria were assessed either by a physician, in baseline data collection or from the data in medical records of patients. Patients excluded from the study were referred to a provincial hospital for appropriate management.

### Ethics consideration

This study was reviewed and approved by the Royal Thai Army Medical Department Institutional Review Board. Written informed consent was obtained from the participants following the WMA Declaration of Helsinki Ethics principles for medical research involving human subjects (approval number: S073q/61_Exp). The study was registered in the Thai Clinical Trials Registry and obliged to disclose details of the 24 mandatory items of the WHO International Clinical Trials Registry Platform (Trial identification number was TCTR20200312007, First submitted date: 12/03/2020).

### Baseline assessment

The allocation was not randomized. At baseline, the participants were voluntarily allocated to intervention and control groups. Face-to-face interviews using standardized questionnaires were conducted in July 2019 at Nong-Hoi Health Promoting Hospital (primary care unit) in Chiang Mai to collect baseline information. The questionnaires included general characteristics, consisting of age, sex, marital status, comorbidities, smoking status, alcohol consumption and HRQoL. The HRQoL was assessed using the Euro-Qol 5 Dimensions 5 Levels (EQ-5D-5L) questionnaire, a standardized measure of health status (Thai version) [14]. This study was permitted to use the EQ-5D-5L questionnaires by the EuroQol Research Foundation (ID Number 28208). The EQ-5D-5L defines health in terms of five dimensions: 1) mobility, 2) self-care, 3) usual activities, 4) pain/discomfort and 5) anxiety/depression [15, 16]. Each dimension has five levels including extreme, severe, moderate, slight and no problem [15, 17]. The health states were converted to utility scores [14]. Additionally, the participants reported a history of forgetting to take their medication in the previous month. Body weight and height were measured using a body composition monitor (OMRON model HBF-212, Kyoto, Japan) and stadiometer (DETECTO, St. Webb City, MO, USA), respectively. Body mass index (BMI) was calculated as weight in kilograms by height in meters squared. Blood pressure (BP) was measured using an automatic BP monitor (OMRON, HEM-7120, Kyoto, Japan) by an operator trained in standardized technique following the 2019 Thai treatment guidelines of HT [18]. The participants were instructed to be stationary at least 5 min in a chair, with feet on the floor and arms supported at the heart level. Two measurements were taken, and the average was recorded. Participants with systolic BP < 140 mmHg and diastolic BP < 90 mmHg were defined as controlled BP [18].

### Intervention

The intervention was established by the model development of primary care services using an innovative network of homecare providers (WinCare) consisting of two main components. The former comprised home care providers, and the latter consisted of a mobile application known as “WinCare” application.

WinCare providers involved novel human resources in the community who were not village health volunteers on duty formerly. The qualification of the home care providers consisted of (i) people in Nong-Hoi Community aged at least 20 years, (ii) holding their highest formal education at least grade 9, (iii) having a motorcycle for transportation, (iv) proficient communication skills, (v) health certified by a physician (vi) able to use a smartphone and mobile application, (vii) without history of criminality certified by the Criminal Records Division, the Royal Thai Police and (viii) without history of illicit drugs used 6 months previously.

**Fig. 1:**
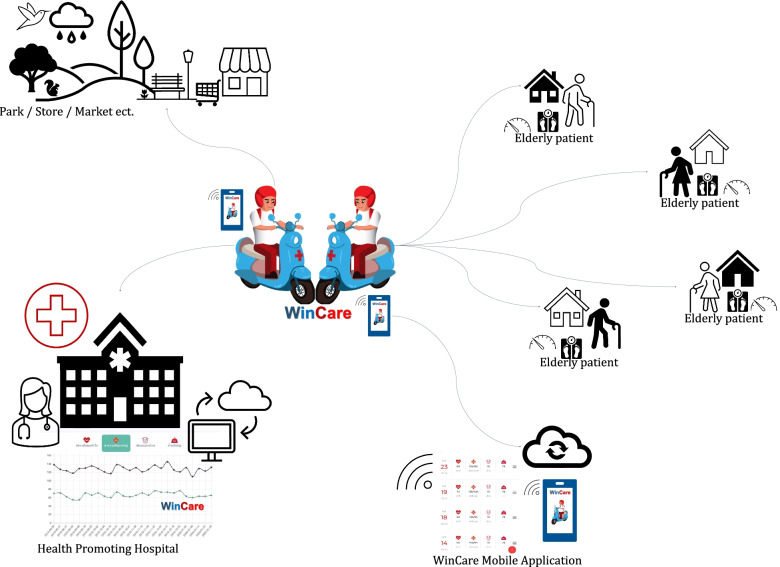
Model development to improve primary care services using an innovative network of homecare providers (WinCare)

WinCare providers were trained and practiced using automatic BP measure and an electronic weighting apparatus. The essential topics included the NCDs and behavioral risk factors (2 h), general care for the elderly with NCDs including T2D and HT (2 h) [18, 19]. Furthermore, WinCare providers were trained to use the automatic BP measurements following the 2019 Thai treatment guidelines of HT [18]. WinCare providers had to independently perform BP measurement and were evaluated by physicians to certify that WinCare providers could use the equipment and appropriately measure BP. Furthermore, training sessions were provided at one and three months. WinCare providers collaborated within their network by demonstrating their willingness as two to three providers per group. An electronic weighing apparatus and automatic BP monitor were provided to each group. WinCare providers were assigned to check-in visits of elderly patients at least once weekly to measure BP and body weight until 6 months. Furthermore, WinCare providers may have played the roles of other home care providers including providing medication reminders, staying active, preparing meals, supplying groceries, arranging transportation, offering companionship, organizing respite care and permitting family caregivers to take breaks (Fig. 1).

The WinCare mobile application (WinCare app) was launched and could be used on a smartphone with both iOS and Android operating systems. WinCare app was designed by the cooperation of the investigators, home care providers and health care workers of Nong-Hoi Health Promoting Hospital to be suitable for use. The Wincare app was used to link among patients, WinCare providers, health promoting hospital and investigators to monitor and enhance effective home care. The WinCare app comprised five main components including (i) the demographic data of patients (ii) contact information of their relatives, (iii) list of all medications and details of administration for patients, (iv) a notification system for taking medication and making doctors’ appointments and (v) health status (BP, pulse rate, body weight and BMI). The health status could be illustrated by graph and interpreted (such as normal BP, high BP, underweight, normal weight, overweight and obese status) to show the trends of the patient’s health outcomes. In addition, updated knowledge about managing patients with HT or T2D such as modifying lifestyle, exercising and dietary behaviors was continuously provided in the WinCare app.

At baseline, the patients in the intervention group received knowledge about the principles of HT and T2D including behavioral risk factors, complications as well as lifestyle modification according to the Thai National Guidelines for Hypertension and Diabetes [18, 19]. Additionally, WinCare providers provided check-in visits to measure their patients, BP and body weight (once weekly), played the roles of other home caregivers for their patients, and recorded measurements and activities in the WinCare app for 6 months. The patients, WinCare providers and health care workers at the primary care unit could access the health status data to remind about health outcomes including BP, BMI and other features such as taking medication and making doctors’ appointments. Therefore, the physician could use the longitudinal data of these patients to appropriately manage treatment.

### Control

At baseline, the patients were included in the study to collect baseline measurements. Similarly, patients in the control group received knowledge about the principles of HT and T2D including complications, behavioral risk factors as well as lifestyle modification according to the Thai National Guidelines for Hypertension and Diabetes [18, 19]. The control group would access standard care for their conditions as usual. Then the outcomes in the control group were collected at one, three and six months.

### Outcomes

The primary outcomes of the study were differences in systolic BP, diastolic BP and controlled BP at six-month follow-up between the intervention and control groups, adjusting for age, sex, marital status, comorbidities, alcohol consumption and smoking status. Secondary outcomes (also adjusted for baseline) included systolic BP, diastolic BP and controlled BP at one- and three-month follow-ups. In addition, HRQoL, BMI and a history of forgetting to take medication at one, three and six months were measured.

### Statistical analysis

Statistical analyses were performed using StataCorp, 2021, *Stata Statistical Software: Release 17,* College Station, TX, USA: StataCorp LLC. Demographic data of participants were analyzed using descriptive statistics. Baseline characteristics of participants between intervention and control groups were compared using the *chi*-square test and *t*-test as appropriate.

We compared the outcome measurements within groups at baseline and at one, three and six months follow-up. For continuous data, including systolic BP, diastolic BP, BMI and HRQoL, repeated measures analysis of variance (ANOVA) was used to compare the change. Trends in controlled BP and a history of forgetting take medication were analyzed using *Chi*-square for trend. To compare outcome measurements between intervention and control groups at baseline and at one, three and six months follow-up, the *chi*-square test and *t*-test were used as appropriate.

To investigate the primary outcome, general linear modelling was used to compare systolic BP, diastolic BP, HRQoL and BMI between the intervention and control groups at follow-up, adjusting for age, sex, marital status, comorbidities, alcohol consumption and smoking status. Additionally, multiple logistic regression analysis was used to determine the magnitude of associations between intervention and binary outcomes (controlled BP and a history of forgetting to take medication). Adjusted odds ratio (AOR) was presented with corresponding 95% confidence intervals (CI). Statistical significance was considered for *p*-value less than 0.05.

## Results

A total of 104 subjects were initially recruited. Of these, six were subsequently excluded from the study after evaluating baseline data for exclusion criteria by the data management unit: four (in the control group) did not provide any data at baseline evaluation including HRQoL and two did not provide written informed consent.

Of the remaining 98 individuals, 52 had been allocated to the intervention group and 46 to the control group. At the first follow-up 1 month after baseline), 98 (100%) individuals (52 in the intervention and 46 in the control group) completed the assessment. At 3 months after baseline, 97 (98.9%) individuals (51 in the intervention and 46 in the control group) attended the evaluation. At 6 months after baseline 94 (95.9%) provided follow-up data.

At baseline assessment, no statistically significant differences were observed between the intervention and control groups regarding age, sex, marital status, comorbidities, a history of forgetting to take medication, controlled BP, systolic BP, diastolic BP, HRQoL and BMI (Table 1). The average age of participants was 69.2 ± 7.2 years. In all, 60 (61.2%) participants were females. The average SBP and DBP of participants were 137.8 ± 16.7 and 76.5 ± 9.6 mmHg, respectively.

**Table 1:** Baseline characteristics of participants

Baseline characteristics	Total	Control	Intervention	***p***-value
	n (%)	n (%)	n (%)	
**n**	98	46	52	
**Age (years)**				
mean ± SD	69.2 ± 7.2	68.4 ± 7.8	70.0 ± 6.6	0.263^†^
**Female**	60 (61.2)	27 (58.7)	33 (63.5)	0.629^¶^
**Marital status**				0.619^¶^
Married	62 (65.3)	29 (67.4)	33 (63.5)	
Widow	25 (26.3)	11 (25.6)	14 (26.9)	
Divorce	6 (6.3)	3 (7.0)	3 (5.8)	
Single	2 (2.1)	0 (0.0)	2 (3.8)	
**Type 2 Diabetes**	40 (40.8)	20 (43.5)	20 (38.5)	0.614^¶^
**Hypertension**	86 (87.8)	42 (91.3)	44 (84.6)	0.313^¶^
**Smoking Status**				0.835^¶^
Never	68 (69.4)	33 (71.7)	35 (67.3)	
Current smoker	6 (6.1)	3 (6.5)	3 (5.8)	
Ex-smoker	24 (24.5)	10 (21.7)	14 (26.9)	
**Alcohol consumption**				0.313^¶^
Never	55 (56.1)	29 (63.0)	26 (50.0)	
Current drinking	17 (17.4)	8 (17.4)	9 (17.3)	
Ex-drinking	26 (26.5)	9 (19.6)	17 (32.7)	
**A history of forgetting to take medication**	29 (29.6)	13 (28.3)	16 (30.8)	0.786^¶^
**Systolic blood pressure (mmHg)**				
mean ± SD	137.8 ± 16.7	137.8 ± 15.5	137.8 ± 17.8	0.985^†^
**Diastolic blood pressure (mmHg)**				
mean ± SD	76.5 ± 9.6	77.4 ± 8.8	75.8 ± 10.2	0.421^†^
**SBP < 140 mmHg and DBP < 90 mmHg**	52 (53.1)	27 (58.7)	25 (48.1)	0.293^¶^
**Body mass index (kg/m**^**2**^**)**				
mean ± SD	25.0 ± 14.4	24.7 ± 3.9	25.3 ± 4.5	0.488^†^
**EQ-5D-5L (HRQoL)**				
mean ± SD	0.903 ± 0.129	0.882 ± 0.151	0.921 ± 0.104	0.138^†^

*SD* standard deviation, *SBP* systolic blood pressure, *DBP* diastolic blood pressure, *HRQoL*, health related quality of life

^†^*t*-test, ^¶^*chi*-square test

Table 2 presents the outcomes within groups at baseline, one-, three- and six-month follow-up. Average systolic BP decreased among patients in the intervention group from 137.8 ± 17.8 mmHg at baseline to 131.1 ± 10.5 mmHg, 129.1 ± 11.8 mmHg and 130.7 ± 11.1 mmHg, at one, three and six months, respectively (*p*-value = 0.018). However, a significant change was not found in average diastolic BP of patients in the intervention group. A significant increase in trends of controlled BP among patients in the intervention group from baseline to 6 months follow-up was observed (*p* for trend = 0.001). Furthermore, in the intervention group, a significant change in average HRQOL was observed (*p*-value = 0.042). In the control group, no differences were found of systolic BP and diastolic BP from baseline to 6 months follow-up. Furthermore, the trends of controlled BP among patients in the control group were also not observed.

**Table 2:** Outcome measurements within groups at baseline and at one, three and six months follow-up

Outcome	Intervention				***p***-value	Control				***p***-value
	Baseline	1-month	3-months	6-months		Baseline	1-month	3-months	6-months	
**Systolic BP (mmHg)**	137.8 ± 17.8	131.1 ± 10.5	129.1 ± 11.8	130.7 ± 11.1	0.018^¥^	137.8 ± 15.5	131.9 ± 11.9	132.9 ± 14.3	133.5 ± 12.5	0.128^¥^
**Diastolic BP (mmHg)**	75.8 ± 10.2	75.0 ± 7.9	74.4 ± 7.9	74.2 ± 6.8	0.313^¥^	77.4 ± 8.8	75.7 ± 9.1	76.3 ± 9.6	79.4 ± 7.8	0.078^¥^
**SBP < 140 mmHg and DBP < 90 mmHg, n (%)**	25 (48.1)	42 (80.8)	40 (78.4)	42 (84.0)	0.001^¶^	27 (58.7)	33 (71.7)	32 (69.6)	29 (65.9)	0.722^¶^
**EQ-5D-5L (HRQoL)**	0.921 ± 0.104	0.906 ± 0.144	0.931 ± 0.119	0.956 ± 0.078	0.042^¥^	0.882 ± 0.151	0.908 ± 0.176	0.912 ± 0.191	0.901 ± 0.169	0.352^¥^
**Body mass index (kg/m**^**2**^**)**	25.3 ± 4.5	25.6 ± 4.6	25.3 ± 4.5	25.4 ± 4.5	0.321^¥^	24.7 ± 3.9	24.5 ± 3.1	24.5 ± 3.8	24.9 ± 3.4	0.362^¥^
**A history of forgetting to take medication, n (%)**	16 (30.8)	7 (13.5)	1 (2.0)	4 (8.0)	0.002^¶^	13 (28.3)	12 (26.1)	10 (21.7)	8 (18.2)	0.224^¶^

*SBP* systolic blood pressure, *DBP* diastolic blood pressure, *HRQoL* health related quality of life

^¥^Repeated measures ANOVA, ^¶^*Chi*-square for trend

Table 3 demonstrates the comparison outcome between intervention and control groups. Average systolic BP of patients in intervention and control groups did not differ at one, three and six months follow-up. However, average diastolic BP of patients in the intervention group (74.2 ± 6.8 mmHg) was relatively low, compared with that in the control group (79.4 ± 7.8 mmHg) at 6 months follow-up (*p*-value = 0.007). No difference was observed of controlled BP among patients between the two groups at one and three months follow-up. However, at 6 months follow-up, controlled BP among patients in the intervention group was 84.0% which was significantly higher than that in the control group (65.9%), (*p*-value = 0.042).

**Table 3:** Comparing outcome measurements between intervention and control groups at baseline and at one, three, and six months follow-up

Analysis	Baseline		***p-value***	1-month		***p-value***	3-months		***p-value***	6-months		***p-value***
	Intervention	Control		Intervention	Control		Intervention	Control		Intervention	Control	
**Systolic BP (mmHg)**	137.8 ± 17.8	137.8 ± 15.5	0.985^†^	131.1 ± 10.5	131.9 ± 11.9	0.777^†^	129.1 ± 11.8	132.9 ± 14.3	0.154^†^	130.7 ± 11.1	133.5 ± 12.5	0.256^†^
**Diastolic BP (mm Hg)**	75.8 ± 10.2	77.4 ± 8.8	0.421^†^	75.0 ± 7.9	75.7 ± 9.1	0.678^†^	74.4 ± 7.9	76.3 ± 9.6	0.289^†^	74.2 ± 6.8	79.4 ± 7.8	0.007^†^
**Controlled BP, n (%)**	25 (48.1)	27 (58.7)	0.293^¶^	42 (80.8)	33 (71.7)	0.292^¶^	40 (78.4)	32 (69.6)	0.319^¶^	42 (84.0)	29 (65.9)	0.042^¶^
**EQ-5D-5L (HRQoL)**	0.921 ± 0.104	0.882 ± 0.151	0.138^†^	0.906 ± 0.144	0.908 ± 0.176	0.974^†^	0.931 ± 0.119	0.912 ± 0.191	0.553^†^	0.956 ± 0.078	0.901 ± 0.169	0.044^†^
**Body mass index (kg/m**^**2**^**)**	25.3 ± 4.5	24.7 ± 3.9	0.488^†^	25.6 ± 4.6	24.5 ± 3.1	0.198^†^	25.3 ± 4.5	24.5 ± 3.8	0.343^†^	25.4 ± 4.5	24.9 ± 3.4	0.575^†^
**A history of forgetting to take medication, n (%)**	16 (30.8)	13 (28.3)	0.786^¶^	7 (13.5)	12 (26.1)	0.115^¶^	1 (2.0)	10 (21.7)	0.002^¶^	4 (8.0)	8 (18.2)	0.140^¶^

*BP* blood pressure, *HRQoL* health related quality of life

^†^*t*-test, ^¶^*Chi*-square test

### Primary outcomes

After adjusting baseline data, no significant difference between baseline average systolic BP and Systolic BP at one, three and six months was observed. However, diastolic BP of patients in the intervention group was on average 5.19 mmHg lower compared with that in the control group at 6 months follow-up (95% CI -8.22, − 2.17). Furthermore, compared with patients in the control group, those in the intervention group were more likely to control BP, (AOR 3.03; 95% CI 1.02–9.01) at 6 months follow-up (Tables 4 and 5).

**Table 4:** Adjusted difference of mean outcomes at one, three and six months follow-up

Outcomes	Groups	1-month	***p***-value	3-months	***p***-value	6-months	***p***-value
		Adjusted difference^**a**^		Adjusted difference^**a**^		Adjusted difference^**a**^	
**Systolic BP (mmHg)**	**Control**	Ref.		Ref.		Ref.	
	**Intervention**	−0.07 (−4.54 to 4.40)	0.975	−3.74 (−9.25 to 1,76)	0.180	−2.81 (−7.72 to 2.08)	0.256
**Diastolic BP (mmHg)**	**Control**	Ref.		Ref.		Ref.	
	**Intervention**	−0.44 (−4.01 to 3.13)	0.806	−1.55 (−5.11 to 2.00)	0.388	−5.19 (−8.22 to − 2.17)	0.001
**EQ-5D-5L (HRQoL)**	**Control**	Ref.		Ref.		Ref.	
	**Intervention**	0.001 (−0.067 to 0.069)	0.973	0.017 (−0.050 to 0.0840)	0.601	0.048 (−0.009 to 0.105)	0.096
**Body mass index (kg/m**^**2**^**)**	**Control**	Ref.		Ref.		Ref.	
	**Intervention**	1.08 (−0.49 to 2.67)	0.173	0.62 (−1.09 to 2.34)	0.474	0.42 (− 1.21 to 2.05)	0.611

*BP* blood pressure, *HRQoL* health related quality of life, *Ref* reference

^a^Mean difference (95% confidence interval) adjusting for age, sex, marital status, comorbidities, alcohol consumption and smoking status

**Table 5:** Magnitude of association between intervention and outcomes at one, three and six months follow-up

Outcomes	Groups	1-month	***p***-value	3-months	***p***-value	6-months	***p***-value
		Adjusted OR^**a**^		Adjusted OR^**a**^		Adjusted OR^**a**^	
**Controlled BP**	**Control**	Ref.		Ref.		Ref.	
	**Intervention**	1.37 (0.48–3.91)	0.562	1.90 (0.70–5.11)	0.202	3.03 (1.02–9.01)	0.046
**A history of forgetting to take medication**	**Control**	Ref.		Ref.		Ref.	
	**Intervention**	0.37 (0.12–1.15)	0.085	0.02 (0.01–0.28)	0.003	0.10 (0.01–0.76)	0.026

*BP* blood pressure

^a^Adjusted odds ratio (95% confidence interval) adjusting for age, sex, marital status, comorbidities, alcohol consumption and smoking status

### Secondary outcomes

From baseline to 6 months follow-up, compared with patients in the control group (0.901 ± 0.169), the HRQoL of those in the intervention group (0.956 ± 0.078) was relatively high (*p*-value = 0.044). Nevertheless, no association was observed in the intervention group for improving HRQoL at one, three and six months follow-up, after adjusting for potential confounders. In terms of average BMI within groups, no significant change was found from baseline to 6 months follow-up in both intervention and control groups. Moreover, the average BMI of patients in the intervention and control groups did not differ at one, three, and six months follow-up.

Additionally, we found a decrease in the trend of a history of forgetting to take medication among patients in the intervention group *(p* for trend = 0.002) while that trend was not observed in the control group. Comparing between two groups, a history of forgetting to take medication among patients in the intervention group was lower at 3 months follow-up (*p*-value =0.002). Nonetheless, differences were not observed of a history of forgetting to take medication among patients in both groups at one and six months follow-up. After adjusting for potential confounders, compared with patients in the control group, those in the intervention group were less likely to have a history of forgetting to take medication, (AOR 0.02; 95% CI 0.01–0.28), (AOR 0.10; 95%CI 0.01–0.76) at three and six months follow-up, respectively (Table 5).

## Discussion

To our knowledge, this constitutes the first study to evaluate the effectiveness of model development of primary care services using an innovative network of homecare providers (WinCare) to reduce BP and improve BP control among elderly patients residing in a suburb, northern Thailand. The outcome suggested that the WinCare intervention could facilitate a reduction of diastolic BP as well as contribute to controlled BP (systolic BP < 140 mmHg and diastolic BP < 90 mmHg) among elderly patients with T2D and/or HT at 6 months follow up. The recent study supported that when the elderly patients whether having HT and/or T2D could maintain their BP at this level, they would be less likely to have an atherosclerotic cardiovascular disease such as stroke [20]. For 6 months, WinCare provided routine BP measurement and weighing for patients at their home once weekly, and then recorded the measured outcomes in the WinCare app. Thus, patients could see their health status including their systolic BP, diastolic BP, weight and BMI as longitudinal data; moreover, the WinCare app would provide feedback of BP results to patients and health care workers at primary care units. Robust evidence confirmed that the home blood pressure monitoring effectively reduced BP [21, 22]. In addition, a recent related study in the UK reported that the digital intervention used in the health context such as BP monitoring and providing feedback of BP results to patients along with optional lifestyle advice led to better control of systolic BP than that of the usual care [23]. Correspondingly, we found that the proportion of patients in the intervention group having a history of forgetting to take medication tended to be lower than that in the control group at three and six months follow-up. These patient compliances may have been encouraged due to receiving social support from WinCare providers, visiting their home every week, talking with and reminding the patients about their medication [24–26]. This positive compliance of the patients may also have enhanced their reduced BP outcomes. Furthermore, the WinCare intervention may serve as one of the service delivery systems comprising essential components of the chronic care model [27, 28]. WinCare intervention providers acted as supporters of the elderly patients to transport and access care between primary care units and patients. Therefore, the elderly patients were able to access care anytime they wanted especially at doctor appointments leading to improved BP control [10].

We found that the HRQoL (EQ-5D-5L) of participants in the intervention group was relatively high, compared with that in the control group at 6 months follow-up. This evidence confirmed that the WinCare intervention including the home care providers and WinCare app could support and provide home care to the elderly patients and may have encouraged positive effects in their QoL including mobility, self-care, usual activities, pain/discomfort level and anxiety/depression [15, 16]. The WinCare intervention provided services for elderly patients including providing medication reminders, preparing meals, supplying groceries, arranging transportation or offering companionship which filled the gap in the daily life activities of the patients. The elderly patients may not have needed deluxe diversions, but rather, simply enjoyed relaxing and having fun which were provided by nonmedical supports [29–31]. Furthermore, a related study in the UK demonstrated that the social prescribing users experienced improvements in their mental and physical health and wellbeing [32].

In Thailand, limited evidence of interventions to improve BP control among patients with HT were available [33, 34]; however, Thailand has been rapidly becoming an aging society with an increase in NCDs, especially HT and T2D. Therefore, the continuum of care approach will play a major role to manage NCDs in the community which links patients, healthcare providers and primary care units. To our knowledge, establishing a network of homecare providers (WinCare) was feasible in a community setting. WinCare offered considerable novel services at the community level. This innovative network of homecare providers would bring a new healthcare workforce closer to the elderly patients in the community. Therefore, WinCare providers comprise additional human resources delivering nonmedical care filling the gaps among health care providers, primary care units and patients residing in a community. Moreover, the WinCare providers also contributed new aspects of home interactions which supported those elderly patients living alone to offer them regular companionship and end the cycle of isolation. This innovation could indirectly improve quality of life in other ways. Because the providers were community members, this innovation model contributed rewarding jobs with additional remuneration for human resources in their local communities. In the future, this novel service should be considered to be implemented for elderly patients needing to develop care plans according to patient requirements and preferences. However, the service fee may be paid by a third party such as the universal health coverage scheme [35] under the National Health Security Office or co-payment with the patients. Hence, the cost-effectiveness of the novel model in the primary health care system should be investigated.

The strengths of the study included being a community-based intervention employing a comparison group. The participants in the study were geriatric patients residing in a suburban community and visiting a health promoting hospital, but not tertiary medical centers. Furthermore, both elderly people with HT and T2D who are the greatest proportion when compared with other chronic diseases Thailand were included in the study. Hence, the results are robust for those elderly residing in a community and being treated at a primary care unit who have been continuously increasing in large numbers in Thailand. Regarding the limitations, the allocation was not randomized. At baseline, the participants were voluntarily allocated to intervention and control groups. However, the baseline characteristics of participants between both groups did not differ. Moreover, after adjusting for potential confounders, the effects of the intervention in primary outcomes analysis were able to be presented at 6 months follow-up. Because the participants in the study comprised elderly patients without any severe complications of diseases, the results of our study could not be generalized to the whole country but may reflect the situation of geriatric patients in the context of a Thai suburban area. Therefore, further studies may be conducted in other settings to evaluate intervention outcomes. In addition, the long term effects of the intervention should be investigated.

## Conclusion

In conclusion, this study showed that an innovative network of homecare providers (WinCare) facilitated elderly patients to improve BP control. The WinCare providers, trained and informed their clients regarding health literacy, who then integrated with the mobile app to provide additional human resources for home care which could fill the gaps among health care providers, primary care units and patients residing in a community.

## Data Availability

The datasets generated or analyzed during the current study are not publicly available because the data sets contain confidential information. Thus, due to ethics restrictions concerning the data sets, they are available from the corresponding author upon reasonable request.

## Abbreviations

HRQoL: Health-related quality of life
NCDs: Noncommunicable diseases
HT: Hypertension
T2D: Type 2 Diabetes
BP: Blood pressure
BMI: Body mass index
